# Shifting beams at normal incidence via controlling momentum-space geometric phases

**DOI:** 10.1038/s41467-021-26406-5

**Published:** 2021-10-18

**Authors:** Jiajun Wang, Maoxiong Zhao, Wenzhe Liu, Fang Guan, Xiaohan Liu, Lei Shi, C. T. Chan, Jian Zi

**Affiliations:** 1 State Key Laboratory of Surface Physics, Key Laboratory of Micro- and Nano-Photonic Structures (Ministry of Education) and Department of Physics, Fudan University, 200433 Shanghai, China; 2 Department of Physics, The Hong Kong University of Science and Technology, Clear Water Bay, Kowloon, Hong Kong China

**Keywords:** Metamaterials, Photonic crystals, Nanophotonics and plasmonics

## Abstract

When hitting interfaces between two different media, light beams may undergo small shifts. Such beam shifts cannot be described by the geometrical optics based on Snell’s law and their underlying physics has attracted much attention. Conventional beam shifts like Goos-Hänchen shifts and Imbert-Fedorov shifts not only require obliquely incident beams but also are mostly very small compared to the wavelength and waist size of the beams. Here we propose a method to realize large and controllable polarization-dependent lateral shifts for normally incident beams with photonic crystal slabs. As a proof of the concept, we engineer the momentum-space geometric phase distribution of a normally incident beam by controlling its interaction with a photonic crystal slab whose momentum-space polarization structure is designed on purpose. The engineered geometric phase distribution is designed to result in a large shift of the beam. We fabricate the designed photonic crystal slab and directly observe the beam shift, which is ~5 times the wavelength and approaches the waist radius. Based on periodic structures and only requiring simple manipulation of symmetry, our proposed method is an important step towards practical applications of beam shifting effects.

## Abstract (web summary)

Light beams can undergo small shifts due to interesting physical phenomena at interfaces. Here, the authors design a photonic crystal slab that enables a large lateral shift at normal incidence by engineering the light-interface interaction through the design of the momentum-space phase distribution.

## Introduction

Light beams are concentrated electromagnetic waves, which go beyond the geometrical descriptions and exhibit extraordinary propagation behaviors. Among them, beam shifts occurring at the interfaces of different optical media during reflection or refraction are well known. Lateral beam shifts in the plane of incidence, resulting from angular dispersion of Fresnel coefficients (which may be different for distinct polarizations), are referred to as Goos–Hänchen (G–H) shifts^1^. Another type of beam shift involving spin–orbit interaction^2–5^ also exists, named as Imbert–Fedorov (I–F) shifts^6,7^. Known as the photonic analog of the spin Hall effect, they are lateral shifts occurring in polarization-dependent directions perpendicular to the plane of incidence^8–12^. Beam shifts in different systems have attracted much attention because of the interesting underlying physics and potential applications^13–18^. However, conventional beam shifts are mostly sub-wavelength and are far smaller than the beam size, and the shifts only happen at oblique incidence. As a result, experimental observations and applications of these beam shifts are hard to realize, especially for I-F shifts^19,20^. A few approaches had been proposed to enlarge the shifts^21–25^, such as implementing ultrahigh-order resonances^26^ and optical bound states in the continuum^27–29^ and utilizing beam-bending metasurfaces^30^. The largest G–H beam shift realized with previous approaches with sub-wavelength structures reaches about 0.4 times the beam waist radius^21^ (where intensity is 1/*e*^2^ the maximum), and it is about 0.1 times for the largest I–F shift^22^. Moreover, oblique incidence are still required in most of previous approaches, and changes in the propagation direction of beams are inevitable.

In this paper, we design a PhC slab without in-plane inversion symmetry to control the momentum-space geometric phase distribution, hence obtaining polarization-dependent lateral beam shifts for a normally incident and outgoing beam. In the experiment, we directly observe a large lateral shift ~5 times the wavelength, approaching the radius of the beam waist. In theory, changing only the inversion-symmetry-breaking parameter of the structure, the obtained beam shift can be adjusted in a wide range. As we will show in the next section, the working principle of our beam shift realization can be applied to all kinds of periodic structures.

## Results and discussion

### Basic principle

For a paraxial light beam, its propagation behavior in free space can be described by its spatial position and propagation direction. Considering a cross plane of a polarized light beam, the projected propagation direction onto the plane corresponds to the expectation value 〈**P**〉 of the in-plane momentum operator $\hat{{{{{{{{\bf{p}}}}}}}}}$, while the beam’s position on the plane corresponds to the expectation value 〈**R**〉 of the in-plane coordinate operator $\hat{{{{{{{{\bf{r}}}}}}}}}$. In most cases, these two expectation values can be studied in the real-space and momentum-space representations. If the considered light beam propagates along the *z* axis, we can formulate its light field (represented as electric field **E**, under decreasing phase convention) on the plane of certain *z* in the aforementioned representations as

1 $$\left|{{{{{{{\bf{E}}}}}}}}({{{{{{{{\boldsymbol{r}}}}}}}}}_{\parallel },z)\right\rangle 	=\left|{{{{{{{\bf{E}}}}}}}}({{{{{{{{\boldsymbol{r}}}}}}}}}_{\parallel },z)\right|{e}^{i\varphi ({{{{{{{{\boldsymbol{r}}}}}}}}}_{\parallel })}\left|\hat{{{{{{{{\bf{E}}}}}}}}}\right\rangle ,\\ \left|{{{{{{{\bf{E}}}}}}}}({{{{{{{{\boldsymbol{k}}}}}}}}}_{\parallel },z)\right\rangle 	=\left|{{{{{{{\bf{E}}}}}}}}({{{{{{{{\boldsymbol{k}}}}}}}}}_{\parallel })\right|{e}^{i\phi ({{{{{{{{\boldsymbol{k}}}}}}}}}_{\parallel })+i{k}_{{{{{{\rm{z}}}}}}}({{{{{{{{\boldsymbol{k}}}}}}}}}_{\parallel })z}\left|\hat{{{{{{{{\bf{E}}}}}}}}}\right\rangle \\ 	\propto \int \left|{{{{{{{\bf{E}}}}}}}}({{{{{{{{\boldsymbol{r}}}}}}}}}_{\parallel },z)\right\rangle {e}^{-i{{{{{{{{\boldsymbol{k}}}}}}}}}_{\parallel }\cdot {{{{{{{{\boldsymbol{r}}}}}}}}}_{\parallel }}{d}^{2}{{{{{{{{\boldsymbol{r}}}}}}}}}_{\parallel }(\,{{\mbox{Fourier transform}}}\,).$$

Here, ***r***_∥_ is the coordinate on the plane, *φ*(***r***_∥_) is the phase of the field at (***r***_∥_, *z*), and $\left|\hat{{{{{{{{\bf{E}}}}}}}}}\right\rangle$ is the polarization state vector of the beam. Meanwhile, ***k***_∥_ is the in-plane projection of free-space momentum ***k***, *ϕ*(***k***_∥_) is the phase of the beam component at certain ***k***_∥_, and ${k}_{{{{{{\rm{z}}}}}}}({{{{{{{{\boldsymbol{k}}}}}}}}}_{\parallel })=\sqrt{{k}^{2}-{{k}_{\parallel }}^{2}}$.

Considering the definition of the in-plane coordinate operator $\hat{{{{{{{{\bf{r}}}}}}}}}$ and momentum operator $\hat{{{{{{{{\bf{p}}}}}}}}}$ and their expectation values, which are detailed in Supplementary Note 1, we can obtain the expressions of 〈**R**〉 and 〈**P**〉 with the above field expressions,

2 $$\langle {{{{{{{\bf{P}}}}}}}}\rangle 	=\left\langle \frac{\partial \varphi ({{{{{{{{\boldsymbol{r}}}}}}}}}_{\parallel })}{\partial {{{{{{{{\boldsymbol{r}}}}}}}}}_{\parallel }}\right\rangle ,\\ \langle {{{{{{{\bf{R}}}}}}}}\rangle 	=-\left\langle \frac{\partial (\phi ({{{{{{{{\boldsymbol{k}}}}}}}}}_{\parallel })+{k}_{{{{{{\rm{z}}}}}}}({{{{{{{{\boldsymbol{k}}}}}}}}}_{\parallel })z)}{\partial {{{{{{{{\boldsymbol{k}}}}}}}}}_{\parallel }}\right\rangle \\ 	={{{{{{{{\bf{R}}}}}}}}}_{{{{{{{{\rm{c}}}}}}}}}-\left\langle \frac{\partial \phi ({{{{{{{{\boldsymbol{k}}}}}}}}}_{\parallel })}{\partial {{{{{{{{\boldsymbol{k}}}}}}}}}_{\parallel }}\right\rangle,$$

where **R**_c_ is a *ϕ*-independent constant. As a pair of reciprocal spaces, the real-space and momentum-space light fields are tightly correlated. Consequently, $< {{{{{{{\bf{R}}}}}}}} >$ and $< {{{{{{{\bf{P}}}}}}}} >$ can be modulated by the phase distributions in their corresponding reciprocal space. Real-space *φ* modulations would cause changes on $< {{{{{{{\bf{P}}}}}}}} >$, changing the propagation direction. Based on this principle, anomalous reflection and refraction of light beams by metasurfaces can be understood as effects of modulated real-space phase gradients^31^. Similarly, if we can modulate the distribution of the momentum-space phase *ϕ*, the modulation on $< {{{{{{{\bf{R}}}}}}}} >$ of the light beam can be realized, i.e., light beams can be shifted in real space. As shown schematically in Fig. 1a, if a phase gradient is introduced to a series of plane waves which compose a beam, the resulting new beam would shift according to the phase gradient in the direction where the additional phase decreases. Considering the mechanism, one can deploy spatial light modulators in the Fourier planes of classical optical paths to artificially shift beams. Conventional light beam shifts, such as the well-known G–H and I–F shifts, can all be attributed to the momentum-space phase gradients induced by reflection or refraction processes at interfaces.

**Fig. 1:**
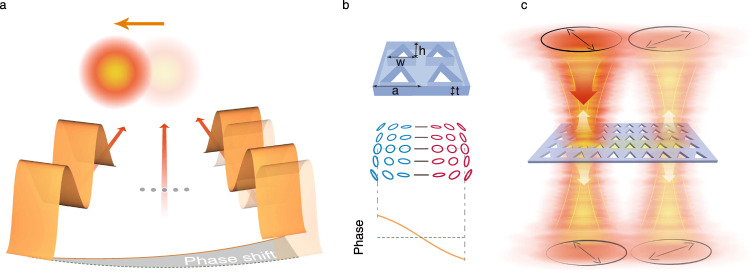
Concept of realizing polarization-dependent lateral shifts of normally-incident Gaussian beams via photonic crystal (PhC) slabs. **a** Schematic view of a beam shift caused by a momentum-space phase gradient. A beam can be viewed as a superposition of plane waves. When an additional phase gradient is introduced to the plane waves in momentum space, the beam will be shifted in the real space in the opposite direction of the gradient. **b** Illustrations of the applied design of a PhC slab which has no in-plane inversion symmetry (upper panel), the corresponding structure of polarization eigenstates near the center of the momentum space (middle panel), and the geometric phase distribution introduced by the PhC slab along the *k*_x_ direction with a $\left|-45\right\rangle$-polarized incident beam (lower panel). **c** Schematic view of a lateral shift realized by the designed PhC slab. A $\left|-45\right\rangle$ polarized beam is normally shined, and after the scattering process, the scattered field can be viewed as four scattered beams: the directly transmitting and reflecting beams and the cross-polarization-converted transmitting and reflecting beams. The cross-polarization-converted beams will be shifted towards the direction opposite to the momentum-space phase gradient.

Among the conventional beam shifts, I–F shifts are polarization-dependent lateral shifts which are known to result from geometric phase gradients^8–11^. Beams with two specific orthogonal polarizations would have opposite phase gradients, leading to opposite shifting directions. Limited by the material properties of the interface, I–F shifts are exceedingly small. To enlarge and control polarization-dependent beam shifts, one way is to engineer the geometric phase gradients in momentum space by implementing nanophotonic structures. PhC slabs, which have designable radiative modes composing of photonic band states^32–36^, are ideal platforms to perform light modulation in the momentum space^37–40^. For a PhC slab, its radiative resonant modes with different in-plane wave vectors within the light cone can couple with free-space plane waves due to reciprocity. Although the modes have different eigen-wavelengths, their leaky nature leads to finite lifetimes and hence finite peak widths in both wavelength and in-plane wave vector. As a result, a monochromatic finite-sized beam composed of a series of plane waves can excite resonant modes which cover an area in the momentum space, even if there is a mismatch in wavelength. Through the coupling, the incident beam components with certain polarization states will be partially converted into ones of orthogonal polarization states. Wave-vector-dependent geometric phase (Pancharatnam–Berry or P–B phase^41,42^) changes will be induced to the cross-polarized outgoing components by the conversion process, in addition to the resonance-induced phase changes. If we further fix the polarization state of the incident beam and the analyzed polarization state of the outgoing beam to be orthogonal, the relative values of the P–B phase changes will be determined by the intermediate polarization eigenstates of the interacting resonances. One can see that, a momentum-space distribution of polarization eigenstates will introduce a corresponding geometric phase distribution to a beam with a specific polarizing-analyzing process^38–40^. By controlling the polarization states of the incident and analyzed beam, and choosing a well-structured PhC slab, we can engineer the momentum-space phase distribution experienced by the analyzed beam and its gradient.

Here, we show one specific design of a PhC slab to perform momentum-space phase engineering and realize large lateral shifts of normally incident Gaussian beams. It is a freestanding silicon nitride (Si_3_N_4_) PhC slab etched with a square lattice of isosceles-triangle holes, as shown in the upper panel of Fig. 1b. The symmetry is engineered such that the in-plane inversion symmetry is broken and only one in-plane mirror axis is maintained. Accordingly, the radiative resonant modes near the center of its Brillouin zone would have a mirror-symmetric polarization structure, in which there would be a change from left-handed circularly polarized (LCP) state to right-handed circularly polarized (RCP) state along the direction perpendicular to the mirror axis, as schematically plotted in the middle panel of Fig. 1b.

Taking advantage of the designed polarization structure we showed, we here choose the two linear polarization states of which the normalized second Stokes parameters S_2_/S_0_ are equal to ±1, rather than the commonly-chosen circular polarization states, to be the fixed incident and analyzing polarization. We mark these two linear states of polarization as ‘ket’s $\left|+45\right\rangle$ and $\left|-45\right\rangle$, while the corresponding analyzed polarization states are ‘bra’s $\left\langle +45\right|$ and $\left\langle -45\right|$ in order to simplify the notations. With Jones calculus and temporal coupled mode theory, the $\left|\mp 45\right\rangle$-polarized transmittance coefficients of a $\left|\pm 45\right\rangle$-polarized plane wave passing through the PhC slab can be obtained as

3 $${t}_{\left|\pm 45\right\rangle } 	=-\frac{1}{2}({t}_{{{{{{\rm{a}}}}}}}+{t}_{{{{{{\rm{b}}}}}}})\frac{\sqrt{{{{{{S}}}}}_{1}^{2}+{{{{{S}}}}}_{3}^{2}}}{{{{{{S}}}}}_{0}}{e}^{i{{\Delta }}{\phi }_{\left|\pm 45\right\rangle }},\\ {{\Delta }}{\phi }_{\left|\pm 45\right\rangle } 	=\mp \left[\arg ({{{{{S}}}}}_{3}+i{{{{{S}}}}}_{1})-\frac{\pi }{2}\right].$$

Here, *S*_0_, *S*_1_, and *S*_3_ are the zeroth, first, and the third Stokes parameters of the polarization state of the resonant mode, which are ***k***_∥_-dependent. *t*_a,b_ are incidence-independent constants containing the resonant effect. It is clear that the differences in the induced geometric phase ${{\Delta }}{\phi }_{\left|\pm 45\right\rangle }$ of a plane wave with a specific *k*_∥_ only depend on two Stokes parameters S_1_ and S_3_ of the polarization eigenstates of the PhC resonance mode which varies in momentum space. The above equation shows that the polarization field profile in Fig. 1b gives a P–B phase distribution in which a net phase gradient $\left\langle \frac{\partial \phi ({{{{{{{{\boldsymbol{k}}}}}}}}}_{\parallel })}{\partial {{{{{{{{\boldsymbol{k}}}}}}}}}_{\parallel }}\right\rangle$ in the ***k***_*x*_ direction can be obtained, as shown in the lower panel of Fig. 1b. As explained by Eq. (2), this engineered phase gradient can cause a real-space shift of the analyzed beam. For example, a normally incident $\left|\pm 45\right\rangle$ beam would have a negative (positive) *x*-directional shift after coupling with the designed slab and being analyzed by a $\left\langle \mp 45\right|$ polarizer, as illustrated in Fig. 1c.

To be mentioned that the shift can happen even without selecting the specific output polarization. The displacement will be reduced in this case since the transmitted beam is a mixture of both the shifted cross-polarized beam and the unshifted co-polarized beam without the momentum-space geometric phase gradient.

### Simulation results and discussion

**Fig. 2:**
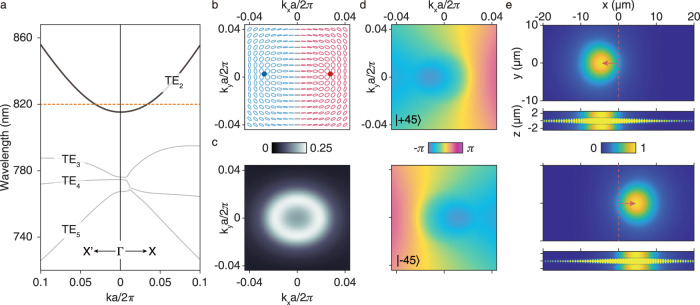
Simulation results of the PhC slab applied to realize polarization-dependent lateral shifts. **a** Simulated transverse-electric-like (TE-like) band structure along the Γ-X and Γ-X' direction. The band we focus on is the second TE-like band (TE_2_), marked by the solid line. We apply this band to realize lateral shift of a normally incident Gaussian beam, in which we take 820 nm as the operating wavelength marked by the orange dashed line. **b** Simulated structure of polarization eigenstates of band TE_2_ in the vicinity of the Γ point. The red (blue) color corresponds to right-handed (left-handed) polarization eigenstates. The red (blue) dots correspond to right-handed (left-handed) circular polarized point. **c** Simulated wave-vector-dependent cross-polarized conversion efficiency between $\left|\pm 45\right\rangle$ polarization states for transmitted light at 820 nm. **d** Simulated wave-vector-dependent phase distribution induced by the $\left|\pm 45\right\rangle$ to $\left|\mp 45\right\rangle$ conversion at 820 nm. Upper plot: $\left|+45\right\rangle$ incidence and $\left\langle -45\right|$ analyzing; lower plot: $\left|-45\right\rangle$ incidence and $\left\langle +45\right|$ analyzing. **e** Top (*z* = −1.5 μm) and cross-section (*y* = 0 μm) view of the realized lateral shifts at 820 nm by simulation. The normally incident Gaussian beam is centered at (*x* = 0, *y* = 0). Only the outgoing beams with converted polarization states are shown by applying analyzing Jones matrices of $\left\langle \mp 45\right|$ to the simulated fields. The color map is normalized according to the maximal intensity of the beam, making the color of the field inside the slab which is enhanced by the guided resonances saturated. To be clear, we mark out the positions of maxima of the shifted beams instead of the centroids. The order of plots are the same as the one of **d**.

To experimentally demonstrate the above approach of realizing lateral shifts, we scaled our design of the freestanding PhC slab to work in the near-infrared spectrum. The thickness *t* of the slab is chosen to be 100 nm, while the period of the etched array is *a* = 660 nm. The height *h* and baseline length *w* of the etched isosceles triangles are equal (*h* = *w* = 550 nm). All the parameters are freely chosen here only considering the ease of fabrication and changes will not affect the occurrence of the beam shifts. Fig. 2a shows the calculated TE-like band of the structure along Γ-X and Γ-X′ directions (*k*_y_ = 0 and *k*_x_ = 0 respectively), where “TE” stands for transverse electric. The far-field polarization eigenstates of the radiative modes on the second TE-like band (TE_2_) are shown in the form of polarization ellipses in Fig. 2b. The red (blue) ellipses correspond to right-handed (left-handed) polarization states, and we marked out the circularly polarized points respectively with corresponding colored dots. It could be seen that the polarization structure and the normalized third Stokes parameters (*S*_3_/*S*_0_) are mirror antisymmetric, and the major axes of the polarization ellipses are mostly horizonal. In other words, the normalized third Stokes parameter of the polarization eigenstates would change from negative to positive in the *k*_x_ direction passing through a linearly polarized line (*S*_3_/*S*_0_ = 0), while the normalized second Stokes parameter (*S*_2_/*S*_0_) is kept near zero, as we expected. This polarization distribution is what we need to induce the momentum-space geometric phase gradients to the beam.

As presented in Fig. 2c, we obtained the ***k***_∥_-dependent cross-polarized conversion efficiency map between the two orthogonal linear polarization states ($\left|+45\right\rangle$ and $\left|-45\right\rangle$) at the wavelength of 820 nm, which determines the divergence angle we would take for the incident beam. Subsequently, the PhC-slab-induced phase differences in momentum space are calculated, as shown in Fig. 2d. One can find that for each case in which $\left|\pm 45\right\rangle$ polarization is converted to $\left|\mp 45\right\rangle$, the geometric phase induced has a trend to decrease in the direction where *S*_3_/*S*_0_ increases, which is consistent with Eq. (3). Furthermore, the momentum-space phase distribution is polarization-dependent: when we exchange the polarizer and analyzer, the phase distribution will be mirror-flipped. Considering the basic principle we introduced, polarization-dependent lateral beam shifts, can be realized. Note that one can observe a phase distortion in each of the calculated phase maps. These phase distortions are caused by dynamical phase shifts induced by the resonances, which are mirror-symmetrical to the *k*_y_-axis. The *x*-direction beam shifts of normally incident beams should only result from the geometric phase distributions due to the mirror symmetry. See Supplementary Note 2 and Note 5 for detailed discussions about resonant phases.

The normally-shined lateral shifts realized by the PhC slab are then confirmed by simulations upon a finite-sized sample consisting of 120 × 120 unit cells. A Gaussian beam of 820 nm is normally shined at the center (*x* = *y* = 0) of the sample. The divergence angle of the beam is chosen to be about 2.5 degrees, covering the high-conversion-efficiency region in the momentum-space as we showed in Fig. 2c. Here in Fig. 2e, we plot the *x*-*y*-plane top views together with the *x* = 0 cross-section views of the simulated light fields. Note that the fields are already cross-polarization analyzed so that only the outgoing beam converted by resonances can be seen in order to view the beam shifts, and the fields without analyzing are included in Supplementary Note 6 for comparison. About 43% of the incident power directly transmits through the slab, 23% of the incident power is directly reflected, 3% is lost due to the finite size of the sample, and about 15% (15%) of the incident power is converted into the transmitted (reflected) cross-polarized beam. One can directly observe that the converted beam is shifted to the left (*x*_peak_ ~−4.6 microns) in the case of a $\left|+45\right\rangle$-polarized incident beam, and to the right (*x*_peak_ ~ 4.6 microns) in the other case ($\left|-45\right\rangle$-polarized). The results agree well with our theoretical prediction that the beam shall shift towards the direction in which the phase decreases, which is controlled by the beam polarization. The calculated beam shift of each case is ~6 times the wavelength, and it is close to the waist radius of the beam (~6 microns). Being large enough, the beam shifts are clearly observable and therefore desirable for application.

Moreover, the applied polarization structure is tunable by changing the geometry of the holes^33^, allowing us to tune the beam shifts. Changing the inversion-symmetry-breaking parameter of the holes, we can modify the momentum-space phase gradient and hence the beam shift continuously. To prove the tunability, We performed simulations in which beam shifts of different displacements are observed. By carefully tuning the polarization structure, the displacement can even exceed the waist radius in theory. The discussions can be found in Supplementary Note 3, Note 4, Note 7, and Note 11, in which we also verified the consistency between the predicted sizes of beam centroid shifts and the simulated ones. Importantly, we only show one specific design according to our designing principle. All flat structures with the same symmetry as the shown one actually have similar momentum-space polarization structures^33–36^, and thus can all be applied to realize similar beam shifts.

### Experimental results and discussion

**Fig. 3:**
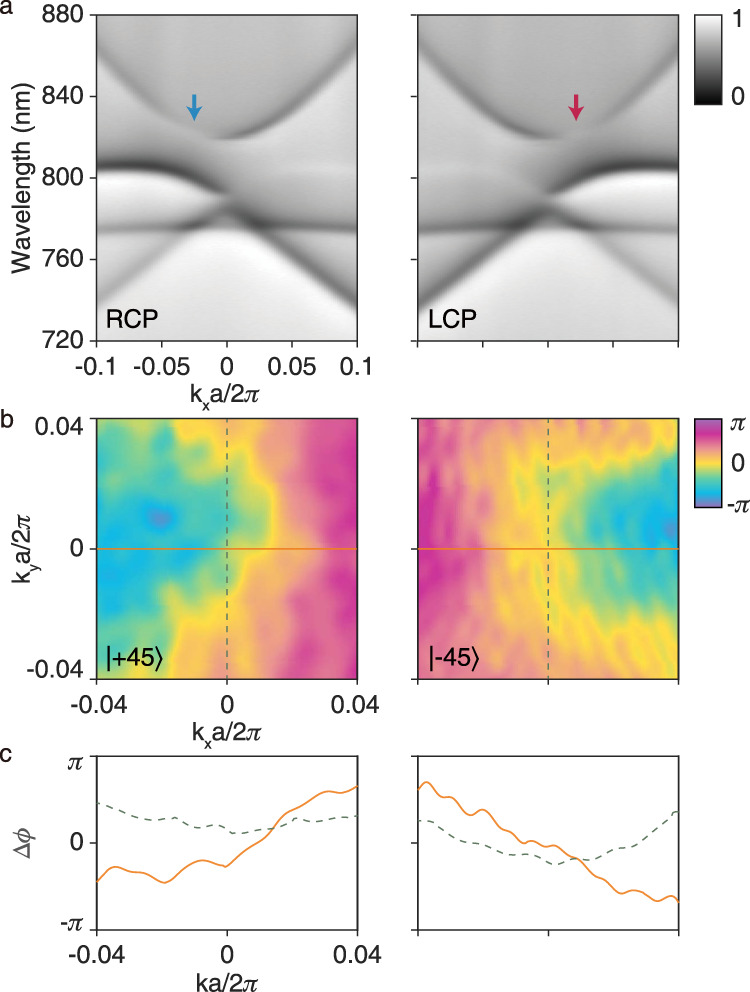
Experimentally measured photonic bands and PhC-induced phase distributions. **a** The measured angle-resolved transmittance spectra of the PhC slab with (left panel) right-handed circularly polarized (RCP) and (right panel) left-handed circularly polarized (LCP) incident beam. Photonic bands of the PhC slab appear as dips in the spectra. The mode with LCP (RCP) polarization eigenstate, marked with blue (red) arrow, will not respond to the RCP (LCP) excitation and turn out to be a diminished point among the band signals. **b** Measured phase distributions induced by the cross-polarized conversion process of the PhC slab at 816 nm. The incident and the analyzed polarizations in the left (right) panel are $\left|+45\right\rangle$ ($\left|-45\right\rangle$) and $\left\langle -45\right|$ ($\left\langle +45\right|$) respectively. **c** The phase distributions along the lines marked in **b**. Solid (dashed) curves in the left and right panels show the phase distributions along the solid (dashed) lines in the left and right panels of **b** correspondingly.

We fabricated the designed PhC slab by etching holes onto a Si_3_N_4_ window on a silicon support frame with the help of electron-beam lithography and reactive-ion etching technique (see “Methods” section for details). The thickness of the window film is about 100 nm, whereas the total number of unit cells is 146 × 146. We applied our home-made Fourier-optics-based momentum-space imaging spectroscopy system^43^ to obtain the polarization-dependent angle-resolved transmittance spectra. Figure 3a shows the measured spectra along Γ-X direction under circularly polarized incidence. The diminished point of the transmittance dips, marked by the red (blue) arrow, corresponds to the radiative mode of right-handed (left-handed) circular polarization on TE_2_ band. The position and handedness of the circularly polarized modes are in accordance with the simulated polarization map. Taking the actual spectra shown in Fig. 3a into consideration, we choose the target wavelength to be 816 nm. Moreover, we experimentally measured the cross-polarized momentum-space phase distributions after interacting with the PhC slab, as shown in Fig. 3b by implementing our phase-measurement system^39^. The measured phase distributions agree well with our simulation results and show expected net phase gradients in *k*_x_ direction.

The real-space beam lateral shifts are then directly observed by using a real-space imaging system (see Supplementary Note 8 for details). Similar to the phase measurement, one linear polarizer is placed on each side of the sample plane to control the incident and analyzed polarizations. The polarization of the incidence polarizer and the analyzer are adjusted to be $\left|+45\right\rangle$ and $\left\langle -45\right|$, respectively ($\left|-45\right\rangle$ and $\left\langle +45\right|$ for the other case). They will not be further rotated in the subsequent measurements to prevent artificial errors. A Gaussian light beam at 816 nm from the tunable laser is then focused on the sample plane at normal incidence, passing through an iris on the front Fourier plane of the focusing lens to constrain the beam’s ***k***_∥_ distribution (divergence angle). The beam waist radius (defined by the position where the intensity is 1/*e*^2^ the maximum) is about 4.1 microns. After analyzed by the analyzer, the intensity distribution of the outgoing beam is captured by a charge-coupled device (CCD), enabling us to directly observe the beam.

**Fig. 4:**
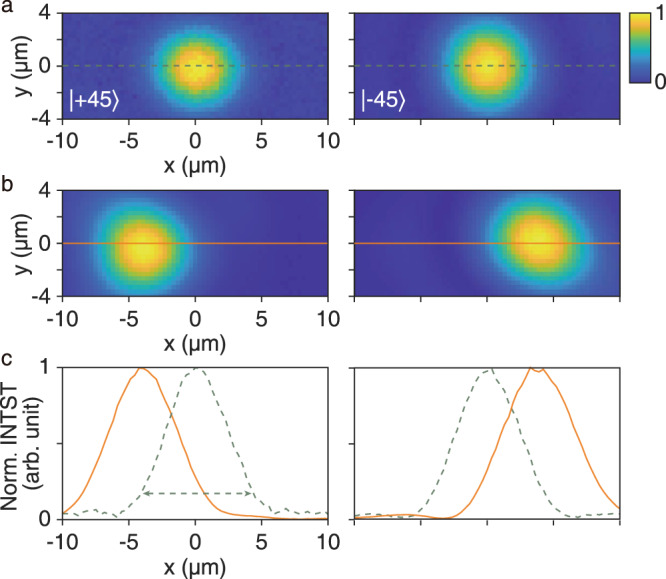
Experimentally observed polarization-dependent lateral shifts. **a** The normalized intensity distributions of the original beam passing through an unstructured Si_3_N_4_ window after the two setups of cross-polarized analyzing. The incident polarization states are marked on the plots, while the analyzed polarizations are orthogonal to the incident polarization. The positions of the beam centers are set as zeros of the coordinates. **b** The normalized intensity distributions of the beam passing through the fabricated PhC slab after the two setups of cross-polarized analyzing. The incident polarization state for each panel is the same as the one in **a**. **c** The normalized intensity distributions of the observed beams along the *x* axis, sliced from **a** and **b**. The dashed green curves correspond to the original beams shown in **a**, whereas the solid orange curves correspond to the shifted beams shown in **b**, correspondingly. The double-headed arrow marks the beam waist.

We first insert an unstructured Si_3_N_4_ window with the same thickness of the PhC slab to the sample plane. Despite the orthogonality of the polarizer and the analyzer, the limited extinction ratio of the polarizers allows a very small portion of the beam to transmit without cross-polarized conversion. We are thus able to locate the original beam and set the zeros of the coordinates. Figure 4a shows the normalized intensity distribution of the transmitted original light beam. Subsequently, we switch the sample to the fabricated PhC slab. As shown in Fig. 4b, the normalized intensity of the cross-polarized beam is measured. Transverse beam shifts can be directly observed. The directions of the beam shifts are consistent with our theoretical predictions. Figure 4c plots the intensity distributions along the lines marked in Fig. 4a, b, from which we can tell that the shift of the intensity peak is about 4.2 microns, agreeing with the estimated shift (about 4.3 microns) from the average phase gradient calculated from the measured phase maps (Fig. 3b). The shifts are clearly observed, proving our proposal. Note that, the overall beam shift without analyzer will be less obvious as only 12% of the incident power is converted into the transmitted cross-polarized beam in the experiment. The results without analyzing are included in the Supplementary Note 9. We also include other results at different wavelengths in the Supplementary Note 10.

We emphasize that our approach to realize large beam shifts is based on nonlocal resonances supported by the PhC slab. This results in the advantage that any domain of the PhC slab can be used for shifting an arbitrary number of beams at the same time. Furthermore, unlike bulky conventional approaches which route light beams using carefully aligned refractors and reflectors, our approach can reposition beams precisely within a few hundreds of nanometers in their propagation direction without changing the direction. This makes the PhC slab quite suitable for routing the light in compact devices.

In conclusion, we have presented the method to realize lateral shifts by utilizing the momentum-space polarization structure of PhC slabs. Momentum-space geometric phase gradients are introduced to light beams via cross-polarized conversion happened in PhC slabs, and large lateral shifts at normal incidence have been experimentally observed. We propose that the momentum-space polarization structure of PhC slabs can work as a new degree of freedom to manipulate light beams. Meanwhile, our results inspire to explore and modulate light beams in real space from the momentum-space perspective.

## Methods

### Theoretical analysis

Please see the Supplementary Information for the detailed derivations and discussions.

### Simulations

The eigenmode simulations and the polarization analysis were performed using a finite-element method. Periodic boundary conditions were applied in the *x* and *y* directions, while the second order scattering boundary condition was applied in the *z* direction. The simulations of the PhC-induced phase and the beam lateral shift were performed by the finite-difference time-domain method. Note that the perfect matching layers were applied for the finite-size simulation. The polarization eigenstates and phase results are obtained from Fourier transformed components of the electromagnetic fields in a periodic unit cell.

### Optical measurements

Please see the Supplementary Note 8 for the measurement setup.

### Sample fabrication

The samples were fabricated on a commercial Si_3_N_4_ window with electron-beam lithography (EBL) and the subsequent reactive-ion etching (RIE) process. The 100-nm-thick Si_3_N_4_ layer resides on a 200-μm-thick center-windowed silicon substrate. For EBL, the raw sample was spin-coated with a layer of positive-tone electron-beam resist (PMMA 950K A4, MicroChem) and an additional layer of conductive polymer (AR-PC 5090.02). The exposure process of EBL was performed by using ZEISS Sigma 300. The sample was then etched by RIE using a mixture of CHF_3_ and O_2_. The EBL-fabricated PMMA layer acts as a mask in the RIE process. After etching, the resist was removed by RIE using O_2_.

## Supplementary information

Supplementary information

## Data Availability

The data that support the plots within this paper and other findings of this study are available from the corresponding authors upon reasonable request.

## References

[1] Goos F, Hänchen H (1947). Ein neuer und fundamentaler versuch zur totalreflexion. Ann. Phys..

[2] O’Neil A, MacVicar I, Allen L, Padgett M (2002). Intrinsic and extrinsic nature of the orbital angular momentum of a light beam. Phys. Rev. Lett..

[3] Allen L, Padgett M, Babiker M (1999). IV the orbital angular momentum of light. Prog. Opt..

[4] Bliokh KY, Rodríguez-Fortuño FJ, Nori F, Zayats AV (2015). Spin–orbit interactions of light. Nat. Photonics.

[5] Eismann J (2020). Transverse spinning of unpolarized light. Nat. Photonics.

[6] Imbert C (1972). Calculation and experimental proof of the transverse shift induced by total internal reflection of a circularly polarized light beam. Phys. Rev. D.

[7] Fedorov FI (1955). K teorii polnogo otrazheniya. Dokl. Akad. Nauk SSSR.

[8] Onoda M, Murakami S, Nagaosa N (2004). Hall effect of light. Phys. Rev. Lett..

[9] Onoda M, Murakami S, Nagaosa N (2006). Geometrical aspects in optical wave-packet dynamics. Phys. Rev. E.

[10] Bliokh KY, Bliokh YP (2006). Conservation of angular momentum, transverse shift, and spin hall effect in reflection and refraction of an electromagnetic wave packet. Phys. Rev. Lett..

[11] Bliokh KY, Aiello A (2013). Goos–Hänchen and Imbert–Fedorov beam shifts: an overview. J. Opt..

[12] Götte JB, Löffler W, Dennis MR (2014). Eigenpolarizations for giant transverse optical beam shifts. Phys. Rev. Lett..

[13] Korger J (2014). Observation of the geometric spin hall effect of light. Phys. Rev. Lett..

[14] Jiang Q-D, Jiang H, Liu H, Sun Q-F, Xie X (2015). Topological imbert-fedorov shift in weyl semimetals. Phys. Rev. Lett..

[15] Kort-Kamp W, Culchac F, Capaz RB, Pinheiro FA (2018). Photonic spin hall effect in bilayer graphene moiré superlattices. Phys. Rev. B.

[16] Chen S, Ling X, Shu W, Luo H, Wen S (2020). Precision measurement of the optical conductivity of atomically thin crystals via the photonic spin hall effect. Phys. Rev. Appl..

[17] Liu Y, Yu Z-M, Xiao C, Yang SA (2020). Quantized circulation of anomalous shift in interface reflection. Phys. Rev. Lett..

[18] Zhu W, Zheng H, Zhong Y, Yu J, Chen Z (2021). Wave-vector-varying pancharatnam-berry phase photonic spin hall effect. Phys. Rev. Lett..

[19] Hosten O, Kwiat P (2008). Observation of the spin hall effect of light via weak measurements. Science.

[20] Qin Y, Li Y, He H, Gong Q (2009). Measurement of spin hall effect of reflected light. Opt. Lett..

[21] Saito H, Neo Y, Matsumoto T, Tomita M (2019). Giant and highly reflective goos-hänchen shift in a metal-dielectric multilayer fano structure. Opt. Express.

[22] Luo H, Zhou X, Shu W, Wen S, Fan D (2011). Enhanced and switchable spin hall effect of light near the Brewster angle on reflection. Phys. Rev. A.

[23] Yin X, Hesselink L, Liu Z, Fang N, Zhang X (2004). Large positive and negative lateral optical beam displacements due to surface plasmon resonance. Appl. Phys. Lett..

[24] Bliokh KY (2016). Spin-hall effect and circular birefringence of a uniaxial crystal plate. Optica.

[25] Kim M (2019). Observation of enhanced optical spin hall effect in a vertical hyperbolic metamaterial. ACS Photonics.

[26] Dai H, Yuan L, Yin C, Cao Z, Chen X (2020). Direct visualizing the spin hall effect of light via ultrahigh-order modes. Phys. Rev. Lett..

[27] Hsu CW, Zhen B, Stone AD, Joannopoulos JD, Soljačić M (2016). Bound states in the continuum. Nat. Rev. Mater..

[28] Koshelev K, Bogdanov A, Kivshar Y (2019). Meta-optics and bound states in the continuum. Sci. Bull..

[29] Wu F (2019). Giant enhancement of the goos-hänchen shift assisted by quasibound states in the continuum. Phys. Rev. Appl..

[30] Yin X, Ye Z, Rho J, Wang Y, Zhang X (2013). Photonic spin hall effect at metasurfaces. Science.

[31] Yu N (2011). Light propagation with phase discontinuities: generalized laws of reflection and refraction. Science.

[32] Fan S, Joannopoulos J (2002). Analysis of guided resonances in photonic crystal slabs. Phys. Rev. B.

[33] Liu W (2019). Circularly polarized states spawning from bound states in the continuum. Phys. Rev. Lett..

[34] Wang J (2020). Routing valley exciton emission of a WS2 monolayer via delocalized Bloch modes of in-plane inversion-symmetry-broken photonic crystal slabs. Light Sci. Appl..

[35] Chen W, Chen Y, Liu W (2020). Line singularities and hopf indices of electromagnetic multipoles. Laser Photonics Rev..

[36] Yoda T, Notomi M (2020). Generation and annihilation of topologically protected bound states in the continuum and circularly polarized states by symmetry breaking. Phys. Rev. Lett..

[37] Guo C, Wang H, Fan S (2020). Squeeze free space with nonlocal flat optics. Optica.

[38] Huang C (2020). Ultrafast control of vortex microlasers. Science.

[39] Wang B (2020). Generating optical vortex beams by momentum-space polarization vortices centred at bound states in the continuum. Nat. Photonics.

[40] Notomi M (2020). Topology in momentum space becomes real. Nat. Photonics.

[41] Berry M (1987). The adiabatic phase and pancharatnam’s phase for polarized light. J. Mod. Opt..

[42] Bomzon Z, Biener G, Kleiner V, Hasman E (2002). Space-variant Pancharatnam–Berry phase optical elements with computer-generated subwavelength gratings. Opt. Lett..

[43] Zhang Y (2021). Momentum-space imaging spectroscopy for the study of nanophotonic materials. Sci. Bull..

