# Supplementary material for: Shifting beams at normal incidence via controlling momentum-space geometric phases
Source: Nat Commun. 2021 Oct 18;12:6046. doi: 10.1038/s41467-021-26406-5 (PMC8523713; doi:10.1038/s41467-021-26406-5)
Supplement: Supplementary file 1 — Supplementary information [file 41467_2021_26406_MOESM1_ESM.pdf]

Shifting beams at normal incidence via controlling momentum-space geometric phases: Supplementary Information

Jiajun Wang[1,*], Maoxiong Zhao[1,*], Wenzhe Liu[1,2,*,†], Fang Guan[1], Xiaohan Liu[1], Lei Shi[1,‡], C. T. Chan[2], and Jian Zi[1,§]

[1]State Key Laboratory of Surface Physics, Key Laboratory of Micro- and Nano-Photonic Structures (Ministry of Education) and Department of Physics, Fudan University, Shanghai 200433, China

[2]Department of Physics, The Hong Kong University of Science and Technology, Clear Water Bay, Kowloon, Hong Kong, China

[*] These authors contributed equally to this work.
[†] wzliu@ust.hk
[‡] lshi@fudan.edu.cn
[§] jzi@fudan.edu.cn

Supplementary Note 1. The detailed basic principle of phase-gradient induced beam shifts

For a paraxial light beam, its propagation behavior in free space can be described by its spatial position and propagation direction. Considering a cross plane of a light beam, the projected propagation direction onto the plane corresponds to the expectation value of the in-plane momentum operator $\hat{\mathbf{p}}$, while the beam's position on the plane corresponds to the expectation value of the in-plane coordinate operator $\hat{\mathbf{r}}$. We here study the expectation values in the real-space representation and the momentum-space representation.

If the considered light beam propagates along the z axis, we are able to formulate its light field (represented as electric field $\mathbf{E}$, under decreasing phase convention) on the cross plane of certain z in the aforementioned two representations as

$$\begin{aligned} |\mathbf{E}(\boldsymbol{r}_\parallel, z)\rangle &= \left|\mathbf{E}(\boldsymbol{r}_\parallel, z)\right| e^{i\varphi(\boldsymbol{r}_\parallel)} |\hat{\mathbf{E}}\rangle, \\ |\mathbf{E}(\boldsymbol{k}_\parallel, z)\rangle &= \left|\mathbf{E}(\boldsymbol{k}_\parallel)\right| e^{i\phi(\boldsymbol{k}_\parallel)+ik_z(\boldsymbol{k}_\parallel)z} |\hat{\mathbf{E}}\rangle \propto \int |\mathbf{E}(\boldsymbol{r}_\parallel, z)\rangle\, e^{-i\boldsymbol{k}_\parallel\cdot\boldsymbol{r}_\parallel} d^2\boldsymbol{r}_\parallel \text{ (Fourier transform).} \end{aligned} \tag{1}$$

Here, $\boldsymbol{r}_\parallel$ is the coordinate on the plane, and the $\varphi(\boldsymbol{r}_\parallel)$ is the phase of the field at $(\boldsymbol{r}_\parallel, z)$. Meanwhile, $\boldsymbol{k}_\parallel$ is the in-plane projection of free-space momentum $\boldsymbol{k}$, $\phi(\boldsymbol{k}_\parallel)$ is the phase of the beam component at certain $\boldsymbol{k}_\parallel$, and $k_z(\boldsymbol{k}_\parallel) = \sqrt{k^2 - {k_\parallel}^2} = \sqrt{k^2 - {k_x}^2 - {k_y}^2}$.

Now, the in-plane position (coordinate expectation, or centroid) $\langle\mathbf{R}\rangle$ and in-plane momentum (momentum expectation) $\langle\mathbf{P}\rangle$ are

$$\langle\mathbf{P}\rangle = \frac{\langle\mathbf{E}|\,\hat{\mathbf{p}}\,|\mathbf{E}\rangle}{\langle\mathbf{E}|\mathbf{E}\rangle}, \quad \langle\mathbf{R}\rangle = \frac{\langle\mathbf{E}|\,\hat{\mathbf{r}}\,|\mathbf{E}\rangle}{\langle\mathbf{E}|\mathbf{E}\rangle}, \tag{2}$$

in which the in-plane coordinate operator $\hat{\mathbf{r}}$ and momentum operator $\hat{\mathbf{p}}$ can be respectively expressed in the real-space and momentum-space representations as

$$\begin{aligned} &\hat{\mathbf{r}} = \boldsymbol{r}_\parallel, \quad \hat{\mathbf{p}} = -i\frac{\partial}{\partial(\boldsymbol{r}_\parallel)}, \quad \text{(real space)} \\ &\hat{\mathbf{r}} = i\frac{\partial}{\partial(\boldsymbol{k}_\parallel)}, \quad \hat{\mathbf{p}} = \boldsymbol{k}_\parallel. \quad \text{(momentum space)} \end{aligned} \tag{3}$$

Putting equation (1) and (3) into (2), we obtain the detailed expressions of $\langle\mathbf{R}\rangle$ and

$$\begin{aligned} \langle\mathbf{P}\rangle &= \left\langle \frac{\partial\varphi(\boldsymbol{r}_\parallel)}{\partial\boldsymbol{r}_\parallel} \right\rangle, \\ \langle\mathbf{R}\rangle &= -\left\langle \frac{\partial(\phi(\boldsymbol{k}_\parallel) + k_z(\boldsymbol{k}_\parallel)z)}{\partial\boldsymbol{k}_\parallel} \right\rangle = \mathbf{R}_\mathrm{c} - \left\langle \frac{\partial\phi(\boldsymbol{k}_\parallel)}{\partial\boldsymbol{k}_\parallel} \right\rangle, \end{aligned}$$

where $\mathbf{R}_\mathrm{c}$ is a ϕ-dependent constant. Clearly, $\langle\mathbf{R}\rangle$ and $\langle\mathbf{P}\rangle$ can be modulated by the phase distributions in their corresponding reciprocal space, i.e., **phase gradients in momentum space can lead to real-space shifts while phase gradients in real space will cause angular changes, as we discussed in the main text.**

Supplementary Note 2. Derivations of the momentum-space geometric phase gradient induced by cross-polarized conversion with PhC slabs

Considering a PhC slab with σ_z-mirror symmetry, we can derive the transmission coefficient matrix for a resonance with specific in-plane wave vector $\mathbf{k}_{||}$ by the temporal coupled mode theory [1, 2]. Here, we only consider frequency range below the diffraction limit of the PhC slab. Hence, the derived transmission coefficient matrix on the $\hat{p}$-$\hat{s}$-polarization basis can be written as

$$\mathbb{F} = \begin{pmatrix} t_{pp} & t_{ps} \\ t_{sp} & t_{ss} \end{pmatrix} = \begin{bmatrix} t - \frac{(t+\alpha_z r)\left|d_p\right|^2}{\gamma - i(\omega-\omega_0)} & -\frac{d_p d_s^*(t+\alpha_z r)}{\gamma - i(\omega-\omega_0)} \\ -\frac{d_s d_p^*(t+\alpha_z r)}{\gamma - i(\omega-\omega_0)} & t - \frac{(t+\alpha_z r)\left|d_s\right|^2}{\gamma - i(\omega-\omega_0)} \end{bmatrix}, \tag{4}$$

where $d_{s,p}$ are the coupling coefficients of the considered Bloch resonance to the free space plane waves, $t_{s,p}, r_{s,p}$ are the transmission and reflection coefficients of the slab without resonances, α_z is the mirror-symmetry factor of the mode (+1 for TE-like mode and −1 for TM-like mode), ω is the oscillating frequency of the system, and ω_0, γ are the real and imaginary part of the considered mode's resonant frequency. No absorption loss is considered and γ only comes from radiation loss.

Now, we have the assumption: the incident beam is normally shined on the sample and the slab resonances all locate in the vicinity of Γ point, therefore $t_x \approx t_y \approx t, r_x \approx r_y \approx r$. The transmission coefficient matrix can be accordingly simplified to be

$$\mathbb{F} = t\mathbf{I} - \frac{t+\alpha_z r}{\gamma - i(\omega-\omega_0)} \begin{pmatrix} \left|d_p\right|^2 & d_p d_s^* \\ d_s d_p^* & \left|d_s\right|^2 \end{pmatrix}. \tag{5}$$

To better describe the interaction between the plane waves with different in-plane momenta and the photonic crystal slab, coordinate transformation is necessary. A rotation matrix shall be introduced which transform (E_p, E_s) to (E_x, E_y) for a plane wave with $\mathbf{k}(\theta, \phi) = (k_0 \sin\theta\cos\phi, k_0 \sin\theta\cos\phi, k_0 \sin\theta)$ $(\theta \approx 0)$:

$$\mathbb{R}(\phi) = \begin{pmatrix} \cos\phi & -\sin\phi \\ \sin\phi & \cos\phi \end{pmatrix}. \tag{6}$$

The polarization state vector $(d_x, d_y)^{\mathrm{T}}$ will hence have the corresponding relation with $(d_p, d_s)^{\mathrm{T}}$:

$$\begin{pmatrix} d_x \\ d_y \end{pmatrix} = \mathbb{R}\begin{pmatrix} d_p \\ d_s \end{pmatrix} = \begin{pmatrix} d_p\cos\phi - d_s\sin\phi \\ d_p\sin\phi + d_s\cos\phi \end{pmatrix}. \tag{7}$$

The $\hat{x} - \hat{y}$ based transmission coefficient matrix shall be transformed from (4) to

$$\tilde{\mathbb{F}} = \mathbb{R}\mathbb{F}\mathbb{R}^{-1} = t\mathbf{I} - \frac{t+\alpha_z r}{\gamma - i(\omega-\omega_0)} \begin{pmatrix} \left|d_x\right|^2 & d_x d_y^* \\ d_y d_x^* & \left|d_y\right|^2 \end{pmatrix}. \tag{8}$$

On the $\left|\pm 45\right\rangle$ basis, the transformed matrix would be

$$\mathbb{F}^{\pm 45} = t\mathbf{I} - \frac{1}{2}\frac{t+\alpha_z r}{\gamma - i(\omega-\omega_0)} \begin{pmatrix} \left|d_x + d_y\right|^2 & (d_x + d_y)(d_x - d_y)^* \\ (d_x - d_y)(d_x + d_y)^* & \left|d_x - d_y\right|^2 \end{pmatrix}. \tag{9}$$

Rewriting this matrix in the form of Stokes parameters [$\mathrm{S}_0 = |d_x|^2 + \left|d_y\right|^2$, $\mathrm{S}_1 = |d_x|^2 - \left|d_y\right|^2$, $\mathrm{S}_2 = 2\mathrm{Re}(d_x d_y^*)$, and $\mathrm{S}_3 = 2\mathrm{Im}(d_x d_y^*)$], we obtain

$$\mathbb{F}^{\pm 45} = t\mathbf{I} - \frac{1}{2}\frac{t+\alpha_z r}{\gamma - i(\omega-\omega_0)} \begin{pmatrix} \mathrm{S}_0 + \mathrm{S}_2 & \mathrm{S}_1 - i\mathrm{S}_3 \\ \mathrm{S}_1 + i\mathrm{S}_3 & \mathrm{S}_0 - \mathrm{S}_2 \end{pmatrix}. \tag{10}$$

If we define $t_a = \frac{1}{2}\frac{(t+\alpha_z r)(\mathrm{S}_0 - \mathrm{S}_2)}{\gamma - i(\omega-\omega_0)}$, $t_b = \frac{1}{2}\frac{(t+\alpha_z r)(\mathrm{S}_0 + \mathrm{S}_2)}{\gamma - i(\omega-\omega_0)}$, and $\Sigma = \frac{1}{2}\arg(\mathrm{S}_3 + i\mathrm{S}_1)$, we can further simplify this matrix into

$$\begin{aligned} \mathbb{F}^{\pm 45} =& t\mathbf{I} - \begin{pmatrix} t_a & 0 \\ 0 & t_b \end{pmatrix} - \frac{1}{2}(t_a + t_b)\frac{\sqrt{\mathrm{S}_1{}^2 + \mathrm{S}_3{}^2}}{\mathrm{S}_0} \begin{pmatrix} 0 & \frac{\mathrm{S}_1 - i\mathrm{S}_3}{\sqrt{\mathrm{S}_1{}^2 + \mathrm{S}_3{}^2}} \\ \frac{\mathrm{S}_1 + i\mathrm{S}_3}{\sqrt{\mathrm{S}_1{}^2 + \mathrm{S}_3{}^2}} & 0 \end{pmatrix} \\ =& \begin{pmatrix} t - t_a & 0 \\ 0 & t - t_b \end{pmatrix} - \frac{1}{2}(t_a + t_b)\frac{\sqrt{\mathrm{S}_1{}^2 + \mathrm{S}_3{}^2}}{\mathrm{S}_0} \begin{Bmatrix} 0 & \exp\left[+i(2\Sigma - \pi/2)\right] \\ \exp\left[-i(2\Sigma - \pi/2)\right] & 0 \end{Bmatrix}. \end{aligned} \tag{11}$$

The first matrix is the co-polarized transmission term. On the other hand, the second term is the cross-polarized conversion term, which consists of three parts: the resonance coefficient $\frac{1}{2}(t_a + t_b)$, the polarization coupling efficiency $\frac{\sqrt{S_1{}^2+S_3{}^2}}{S_0}$, and the geometric phase factor matrix. The geometric phase factor would be $\Delta\phi_{|\pm45\rangle} = \mp\left[\arg(S_3 + iS_1) - \frac{\pi}{2}\right]$.

The geometric phase induced can also be understood with the Poincaré Sphere, which is shown in Supplementary Figure 1. The cross-polarized conversion processes shown above can also be represented by semi-great-circle trajectories on the normalized Poincaré sphere, whose starting, intermediate, and ending points represent the incident, resonant, and analyzed polarization states. When we fix the starting and ending points to be $|\pm45\rangle$ as explained, the differences in the P-B phase induced by two different resonances are only determined by their polarization eigenstates, which equals half the geometric solid angle enclosed by the two corresponding trajectories (see the two arrowed curves in Supplementary Figure 1). The sign of the difference is determined by the direction of the loop formed by the trajectories (positive for counterclockwise, negative for clockwise). With the previous definitions, we can find the solid angle shall be $4\Delta\Sigma$, and thus the geometric phase difference shall be $2\Delta\Sigma$. If we choose a reference trajectory of which $\Sigma = \pi/4$ to be the zero point, the geometric phase of a certain resonance shall be $\Delta\phi_{|\pm45\rangle} = \mp2(\Sigma - \pi/4) = \mp[\arg(S_3 + iS_1) - \pi/2]$, agreeing with the derived one.

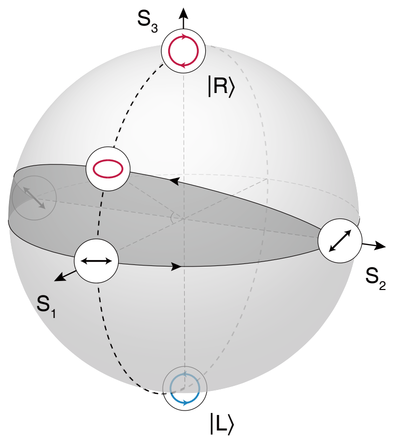

Supplementary Figure 1: **The visual way to understand the induced geometric phase.** On the Poincaré sphere, polarization eigenstates of the PhC slab's resonant modes can be mapped as points, and cross-polarization conversion processes caused by beam-resonance coupling can be viewed as trajectories. S_1, S_2 and S_3 are the first, second and third Stokes parameters, respectively. The shadowed area shows the solid angle enclosed by the two trajectories we choose, which corresponds to two times the difference in the geometric phases induced by the two trajectories.

One can find that the first and the third part of the second term in Eqn. (11) both contribute to the net phase gradient in momentum space. For our system, the geometric phase factor has a net gradient in the k_x direction. On the other hand, the resonance coefficient $t_a + t_b$ is mirror-symmetric about k_x and k_y axes and rotationally symmetric about k_z axis, which follows the symmetry of the system. However, S_3 in the polarization coupling efficiency $S_1{}^2 + S_3{}^2$ is only mirror symmetric about k_y axis since the time-reversal symmetry of the system is broken by open boundary condition (while the reciprocity is maintained). Therefore, there will be no net k_x-gradient resulting from the resonant phase. This will be verified in **Supplementary Note 5**. The k_y-gradient contribution from resonant phase is non-zero, though it is very small in our system.

For the sake of simplicity, in the following parts of the supplementary information we will replace $\mathbb{F}^{\pm45}$ with $\mathbb{F}$ for convenience. $\mathbb{F}$ will no longer represent the original p-s-polarization based transmission coefficient matrix.

Supplementary Note 3 Constructing a quasi-analytic polarization field near Γ point and its geometric phase gradient

In our previous work, we proved that by breaking the in-plane C_2 symmetry of a photonic crystal slab, the central bound states in the continuum on photonic bands will all be broken into pairs of circularly polarized resonances which locate in the $\Gamma - \mathrm{X}$ direction [1]. Based on this principle, we here construct a quasi-analytic polarization field with detailed k-dependent expressions of d_x and d_y to show how the geometric phase gradient can be constructed with the chosen $\left|\pm 45\right\rangle$ basis.

For an original C_{4v} symmetric system, the far-field polarization field of the system would follow the symmetry. We here take the polarization field which corresponds to the A_2 irreducible representation, and apply the lowest-order polynomial approximation, *i. e.* $d_x^{\text{origin}} = i\mu k_y, d_y^{\text{origin}} = -i\mu k_x$. [To be mentioned that we will not take the zeroth-order approximation $(0, 0)$ as the original field since it is an impossible trivial case]. Such a field, which is a linear approximation of a magnetic dipole-moment perpendicular to the sample plane, would have a singular point at $(k_x = 0, k_y = 0)$ corresponding to an original bound state in the continuum. Subsequently, by breaking the mirror symmetry along the k_x direction, a perturbation will be introduced. Eigenvalue of the mirror symmetry about the k_y axis of this perturbation is required to be compatible with the original one (-1), thus the d_x perturbation term is k_x-even while d_y perturbation term is k_x-odd. We also choose the perturbation terms to be the lowest-order polynomials: $(d_x^{\text{perturb}}, d_y^{\text{perturb}}) = (\nu, 0)$. This perturbation is a linear approximation of a electric dipole-moment lying in the sample plane and have a phase difference $\pi/2$ comparing to the original electric one. Therefore the perturbed field would be $(d_x, d_y) = (\nu + i\mu k_y, -i\mu k_x)$. The derivation we made in the previous part is consistent with this field. We can find two circularly-polarized points $(k_x^c, k_y^c) = (\pm\nu/\mu, 0)$ in the field, mimicking a real system well. Now we can directly write down the Stokes parameters,

$$
\begin{aligned}
\mathrm{S}_0 &= |d_x|^2 + \left|d_y\right|^2 = \nu^2 + \mu^2 k_y^2 + \mu^2 k_x^2 \\
\mathrm{S}_1 &= |d_x|^2 - \left|d_y\right|^2 = \nu^2 + \mu^2 k_y^2 - \mu^2 k_x^2 \\
\mathrm{S}_2 &= 2\mathrm{Re}(d_x d_y^*) = 2\mathrm{Re}[(\nu + i\mu k_y) i\mu k_x] = -2\mu^2 k_x k_y \\
\mathrm{S}_3 &= 2\mathrm{Im}(d_x d_y^*) = 2\mathrm{Im}[(\nu + i\mu k_y) i\mu k_x] = 2\mu\nu k_x.
\end{aligned}
\tag{12}
$$

From the expressions we can get the normalized third Stokes parameter $\mathrm{S}_3/\mathrm{S}_0$,

$$
\rho_c = \frac{4k_x\kappa}{4 + (k_x^2 + k_y^2)\kappa^2}, \tag{13}
$$

where κ is defined as $\kappa = 2\mu/\nu$. This factor has the unit of length (or k-space phase gradient), and it is four times the inverse of the distance between the circularly polarized states. At the mean time, we can obtain the k-space geometric phase distribution $\Delta\phi_{|\pm 45\rangle}(\mathbf{k}_{\|})$ together with the k_x phase gradient. Here we just take the $\left|+45\right\rangle$ input & $\left|-45\right\rangle$ output case as the example:

$$
\Delta\phi_{|+45\rangle} = -\arg\{4k_x\kappa + i[4 - (k_x^2 - k_y^2)\kappa^2]\} + \pi/2, \tag{14}
$$

$$
\partial(\Delta\phi_{|+45\rangle})/\partial k_x = \frac{4\kappa[4 + (k_x^2 + k_y^2)\kappa^2]}{16 + 8(k_x^2 + k_y^2)\kappa^2 + (k_x^2 - k_y^2)^2\kappa^4}. \tag{15}
$$

Now we can understand the meaning of κ. For the case $(k_x, k_y) = (0, 0)$, the gradient would be κ, and it is actually the maximum of the momentum-space phase gradient. Note that the resonant phase factor with the polarization coupling term is mirror symmetric about k_y axis, so after an integration symmetric about the k_y axis as we mentioned, its net effect on beam shift in the x direction shall be cancelled. Hence, only the geometric phase contributes to the beam shift in the x direction, and κ is the factor which determines the upper limit of beam shift in the x direction caused by the geometric phase gradient.

Supplementary Note 4 The approximated beam shifts in case of a Gaussian incident beam

Having the expression of the phase gradient and the transmission coefficients, we can study the relation between the perturbation and the beam shift in a numerical way. For a beam before and after interaction with the sample, its real-space position expectation (centroid) can be represented by $\langle \mathbf{R} \rangle = \frac{\langle \mathbf{E}|\hat{\mathbf{r}}|\mathbf{E}\rangle}{\langle \mathbf{E}|\mathbf{E}\rangle}$, where $\hat{\mathbf{r}} = i\partial/\partial\mathbf{k}$. In general non-paraxial beam cases, there would be geometric phase effect due to the variation of the wave vector inside the beam. But, as we showed in the **Supplementary Note 2**, with the incident beam restricted to be normally shined and paraxial, this effect can be neglected. As a result, the position operator can be simplified to $\hat{x} = -\partial\varphi/\partial k_x$ for the x-direction beam shift we concern about, which corresponds to the expectation value of the x-direction phase gradient with a factor of -1. The same for the y direction. The asymmetry of momentum-space polarization field and corresponding geometric phase gradient in the k_y direction may induce y-direction beam shift as well, but this effect is more about the broken time reversal symmetry, which is not our concern here.

We here choose a Gaussian beam which is focused on the sample and of which the beam axis is normal to the sample plane. Such a Gaussian beam would have the k-space field distribution

$$G(k_x, k_y) = \frac{2}{\pi k_{\parallel}^2} e^{-\frac{k_x^2+k_y^2}{k_d^2}}, \tag{16}$$

where k_d is the divergence wave vector of the beam. The divergence angle of the beam would be $\arcsin(k_d/k_0)$, and the beam radius at the waist will be $2/k_d$. Hence the state vector can be written as $|\mathbf{E}) = |\mathbf{e})\, G(k_x, k_y)$, where $\mathbf{e}$ is the Jones vector of the incident beam. If we assume the incident beam is purely $|+45\rangle$-polarized, *i. e.* $\mathbf{e} = (1, 0)^{\mathrm{T}}$, the expected shift of the output cross-polarized beam can be obtained as

$$\begin{aligned}
\Delta \langle \mathbf{R}_{45\to-45} \rangle &= \langle \mathbf{R}_{45\to-45} \rangle - \langle \mathbf{R}_{\mathrm{in}} \rangle \\
&= \frac{\langle \mathbf{E_{in}}| \mathbb{F}^{\dagger}_{anti} \hat{\mathbf{r}} \mathbb{F}_{anti} |\mathbf{E_{in}}\rangle}{\langle \mathbf{E_{in}}| \mathbb{F}^{\dagger}_{anti} \mathbb{F}_{anti} |\mathbf{E_{in}}\rangle} - \frac{\langle \mathbf{E_{in}}| \hat{\mathbf{r}} |\mathbf{E_{in}}\rangle}{\langle \mathbf{E_{in}}|\mathbf{E_{in}}\rangle} \\
&= \frac{1/4 \int |t_a + t_b|^2\, G^2 \frac{\mathrm{S_1}^2+\mathrm{S_3}^2}{\mathrm{S_0}^2} \frac{\partial(\varphi_r+\varphi)}{\partial \mathbf{k}} dk_x dk_y}{1/4 \int |t_a + t_b|^2\, G^2 \frac{\mathrm{S_1}^2+\mathrm{S_3}^2}{\mathrm{S_0}^2} dk_x dk_y} - 0 \\
&= \frac{1/4 \int |t_a + t_b|^2\, G^2 \frac{\mathrm{S_1}^2+\mathrm{S_3}^2}{\mathrm{S_0}^2} \frac{\partial(\varphi_r+\varphi)}{\partial \mathbf{k}} dk_x dk_y}{1/4 \int |t_a + t_b|^2\, G^2 \frac{\mathrm{S_1}^2+\mathrm{S_3}^2}{\mathrm{S_0}^2} dk_x dk_y},
\end{aligned} \tag{17}$$

where $\mathbb{F}_{anti}$ is the anti-diagonal part of the transmission coefficient matrix $\mathbb{F}$ on the $|\pm45\rangle$ degree basis (the second term of Eqn. (11)), and $\varphi_r = \arg(t_a + t_b)$ is the phase shift caused by resonance. As we discussed in last paragraph, the resonant phase is mirror symmetrically distributed about the k_y axis, therefore it will not affect the x-direction beam shift. In order to reduce the number of parameters other than μ and ν (or rather κ), we also remove the dispersion-dependent$|t_a + t_b|^2$ term. As a result, the approximated expression of x-direction shift will be

$$\Delta^{x,\,45\to-45}_{approx.} = \frac{\int G^2 \frac{\mathrm{S_1}^2+\mathrm{S_3}^2}{\mathrm{S_0}^2} \frac{\partial\varphi}{\partial k_x} dk_x dk_y}{\int G^2 \frac{\mathrm{S_1}^2+\mathrm{S_3}^2}{\mathrm{S_0}^2} dk_x dk_y}. \tag{18}$$

Let us take a look at the detailed expressions. The numerator would be

$$\int G^2 \frac{\mathrm{S_1}^2+\mathrm{S_3}^2}{\mathrm{S_0}^2} \frac{\partial\varphi}{\partial k_x} dk_x dk_y = \int \frac{16\kappa e^{-\frac{2(k_x^2+k_y^2)}{k_d^2}}}{k_d^4 \pi^2} \frac{1}{(4 + k_x^2\kappa^2 + k_y^2\kappa^2)} dk_x dk_y \left[= -\frac{16 e^{\frac{8}{k_d^2\kappa^2}} \mathrm{Ei}(-\frac{8}{k_d^2\kappa^2})}{k_d^4 \pi\kappa} \right], \tag{19}$$

while the dominator is not an easy integral:

$$\begin{aligned}
\int G^2 \frac{\mathrm{S_1}^2+\mathrm{S_3}^2}{\mathrm{S_0}^2} dk_x dk_y &= \int \frac{e^{-\frac{2(k_x^2+k_y^2)}{k_d^2}} [64 + 32(k_x^2 + k_y^2)\kappa^2 + 4(k_x^2 - k_y^2)^2\kappa^4]}{k_d^4 \pi^2 (4 + k_x^2\kappa^2 + k_y^2\kappa^2)^2} dk_x dk_y \\
&= \int \left[\frac{4 e^{-\frac{2(k_x^2+k_y^2)}{k_d^2}}}{k_d^4\pi^2} \left(1 - \frac{4k_x^2 k_y^2 \kappa^4}{(4 + k_x^2\kappa^2 + k_y^2\kappa^2)^2}\right) \right] dk_x dk_y.
\end{aligned} \tag{20}$$

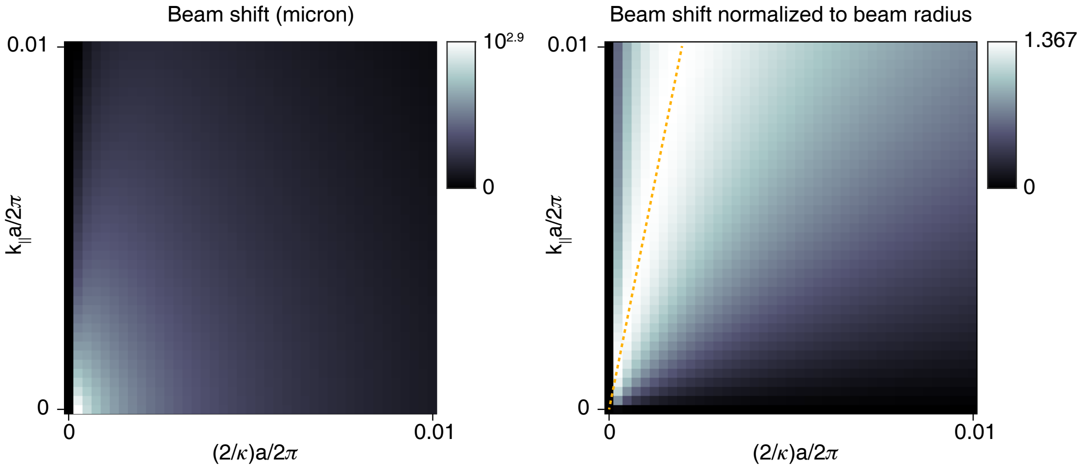

Supplementary Figure 2: **The numerically approximated beam shifts.** (Left) Calculated beam shifts in log scale. It is expected to be approaching infinity when κ is approaching zero. (Right) Calculated beam shifts normalized to the beam radius $2/k_d$. Maxima can be found for different values of κ and k_d, ~ 1.367 times the beam radius.

Instead of analytically computing the integral, we may numerically calculate and visualize it. Setting the normalizing 'period' $a = 660$ nm, one can see the result as Supplementary Figure 2. As expected, the beam shift can approach infinity (which cannot be reached as the $\kappa = 0$ case correspond to a BIC that cannot be coupled with) if we make κ and k_d both approach zero. However, the beam shift shall be compared to the beam radius. **If the beam becomes a plane wave, the beam shift will turn out to be meaningless.** Interestingly, we will find a maximum in normalized beam shifts when we tune the parameter κ and k_d. **The largest beam shift would exceed the beam radius (about 1.367 times of the beam radius).**

Supplementary Note 5. The geometric phase factor and resonance phase factor

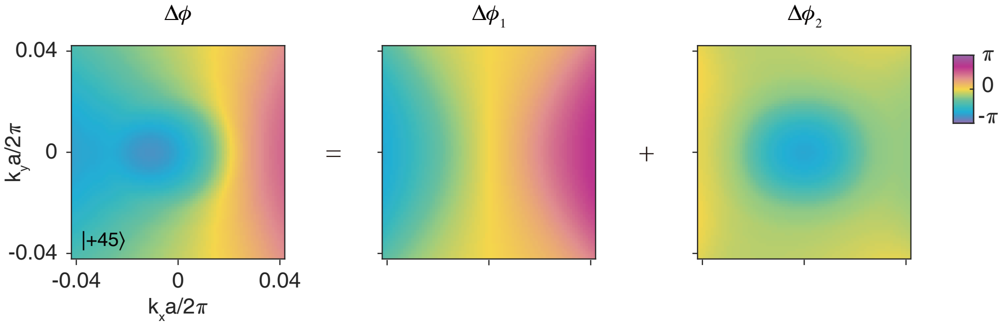

Supplementary Figure 3: **PhC-induced phase distribution consisting of two parts**. $\Delta\phi$ is the PhC-induced distribution with the polarization state of the incident beam and the analyzing polarization chosen to be $|+45\rangle$ and $\langle-45|$ respectively, which has been shown in the second figure in our main text. $\Delta\phi_1$ is the geometric phase induced by the cross-polarized conversion. $\Delta\phi_2$ is the resonance-caused phase.

As discussed in **Supplementary Note 2**, the total phase shift of the beam components after passing through the photonic crystal slab consists of two parts, the geometric phase shift and the resonant phase shift. We here show the results extracted from the simulation, separating the two parts.

Supplementary Note 6. The field profile of the sample without selecting specific output polarization: simulation

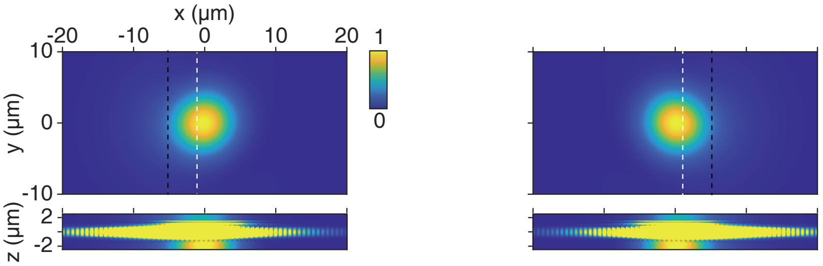

Supplementary Figure 4: The simulated field profile of the sample without analyzing. (Left) $|{+45}\rangle$-polarized incidence, (right) $|{-45}\rangle$-polarized incidence. The black dashed lines correspond to the positions (centroids) of the cross-polarized beams, while the white dashed lines are the positions (centroids) of the beams without analyzing.

In the main text, we show the field profile with the analyzing Jones matrix applied to show the shifted cross-polarized beams clearly. Without analyzing, the beam shifts can still happen, but the displacements will become small as the major parts of the non-analyzed transmitted beams are the co-polarized beams with no net momentum-space phase gradient. Unlike in the main text, we mark the positions of the centroids of the beams which we analyze in theory.

Supplementary Note 7. Modulating the polarization-dependent lateral shifts by controlling the symmetry-breaking factor: simulation

Here we demonstrate the tunability of the polarization-dependent lateral shift we realized. We turn the holes into isosceles trapezoids with different topline-to-baseline ratios. The area of the trapezoid is maintained and we make the height equal to the baseline length ($L = h$). The symmetry-breaking factor β is then defined as the ratio of the reduced topline length $2\Delta L$ to the baseline length L (see Supplementary Figure 5a). Subsequently, we construct different PhC-induced momentum-space phase gradients by changing β. With larger perturbation β, the circularly polarized points in momentum space would get farther from the Γ point, i.e., κ defined in **Supplementary Note 3** would decrease and the phase gradient would correspondingly change. The phase distributions obtained from simulations are shown as Supplementary Figure 5b. The larger the symmetry-breaking factor β is, the smaller the induced phase gradient becomes. As a result, the polarization-dependent lateral shift will decrease with the increasing symmetry-breaking factor. We shine a $|{+45}\rangle$-polarized gaussian beam of which the divergence angle $\sim$ 1.04 degree, covering a momentum-space region whose radius $\sim 0.015\times 2\pi/a$ (a = 660 nm), onto the center of the sample. The longitudinal sections of the simulated field profiles after analyzed by $|{-45}\rangle$-polarization are shown in Supplementary Figure 5c. Different beam shifts can be clearly observed, and with small β's the beam shifts show the possibility of approaching and exceeding the beam radius (14.5 microns). It may seem counterintuitive that in the cases with larger perturbations, the shifts become smaller. This is because the starting non-perturbed case is actually a singularity (BIC) of beam shift here, and the perturbations make the system fall back to a regular one-dimensional grating in which polarization-dependent lateral shift based on $|\pm 45\rangle$ polarizations will not happen.

In Supplementary Figure 5d, we compare the simulated beam displacements with the calculated ones from the phase distributions applying Eqn. (18). A good consistency is shown except for the $\beta = 0.1$ case. The error comes from the very-large phase gradient and a more lumped cross-polarization conversion efficiency, which goes beyond the momentum-space resolution we used in phase simulations.

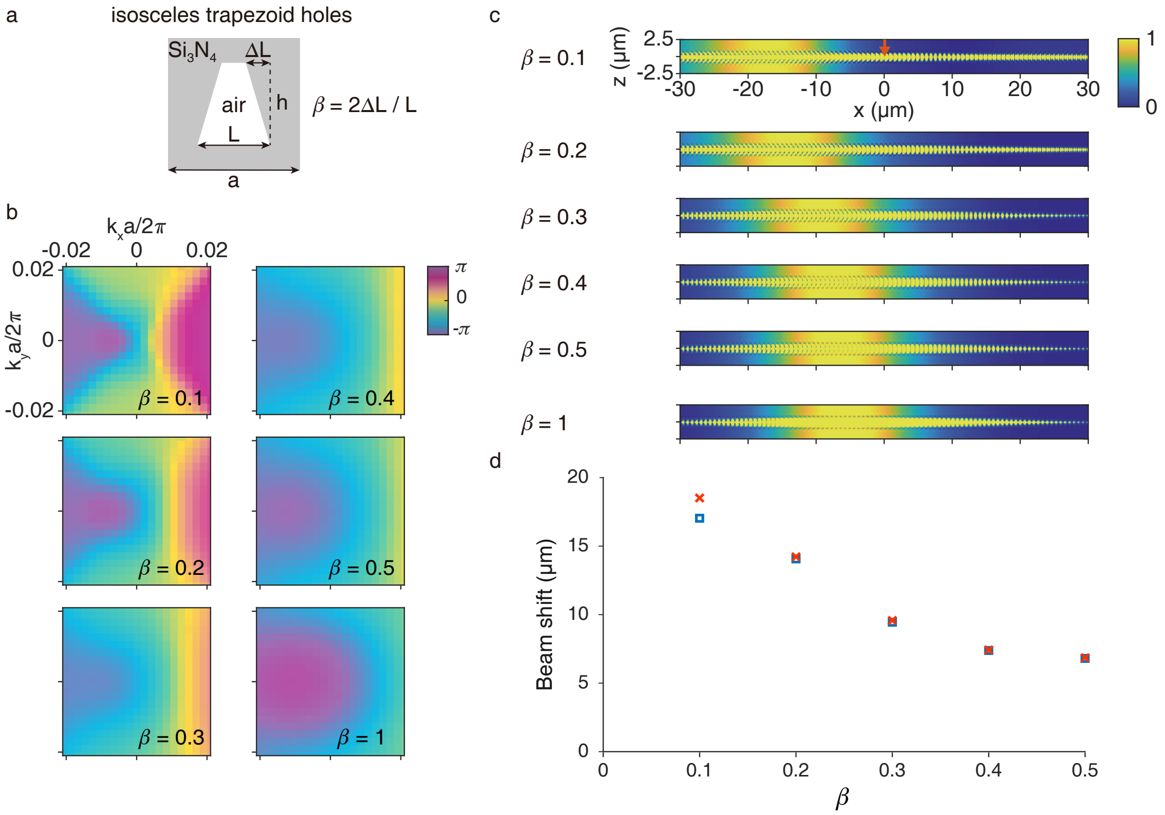

Supplementary Figure 5: **a**, A schematic view of the structure and the definition of the symmetry-breaking factor β. **b**, The different momentum-space phase distributions of structures with different symmetry-breaking factors. The beam is $|45\rangle$-polarized and $\langle -45|$-analyzed. **c**, Longitudinal sections of simulated field profiles, showing different beam displacements in PhC slabs with different symmetry-breaking factors. The $|45\rangle$-polarized incident beam is located at the center of the sample, and the profiles have been $\langle -45|$-analyzed. With a larger symmetry breaking parameter, the phase gradient becomes smaller and results in a smaller beam shift. **d**, The values of beam displacements, obtained from simulations (blue squares) and theoretical calculations (red crosses).

Supplementary Note 8. The measurement setup

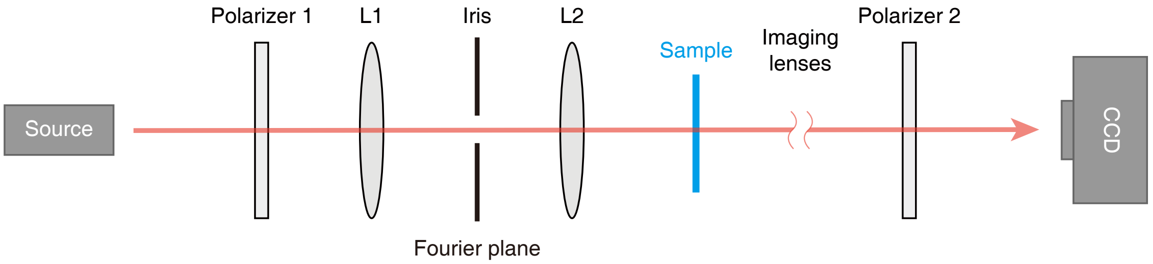

Supplementary Figure 6: **Schematic of the real-space imaging system**. L1 and L2 are lenses.

Here, we show the schematic of our measurement setup. A light beam from a tunable laser is Fourier transformed by lens L1, filtered by an iris which constrains the momentum-space range covered by the beam, and again focused by lens L2. Then it is shined onto the sample at normal incidence. The polarization of the incident beam and the analyzing polarization can be adjusted by rotating the two linear polarizers at the two ends of the light path. Passing through the sample, the outgoing light is finally analyzed and imaged with lenses onto the CCD.

Supplementary Note 9. The field profile of the sample without selecting specific output polarization: experiment

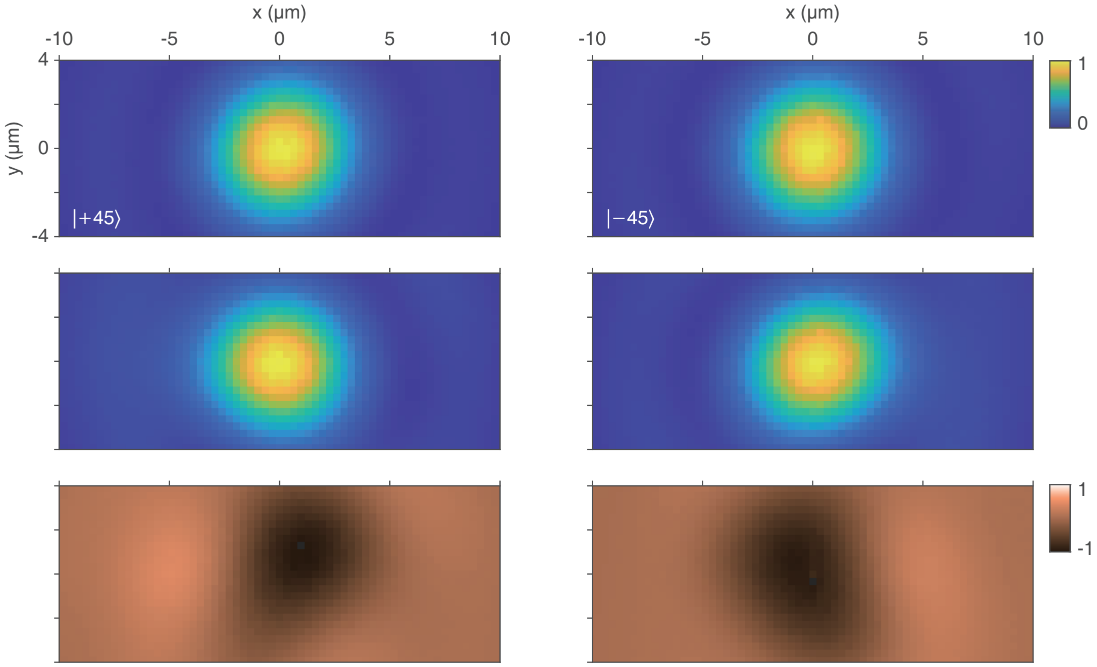

Supplementary Figure 7: **Real-space measurement of the transmitted beam without analyzer at 819 nm. The first row** shows the incident beams with different polarizers. **The second row** shows the output beams without analyzing. **The third row** shows the relative transmission difference maps, which show the slight displacements of the output beams.

As we explained in the main text and **Supplementary Note 6**, the output beams without analyzing are deformed because they are made of both the shifted cross-polarization converted beam and the directly transmitting co-polarized beam. As a result, their displacements are less obvious. But they are truly shifted and we can use the relative transmission difference $[I_{\text{output}}(x,y) - I_{\text{incident}}(x,y)]/\max[\left|I_{\text{output}}(x,y) - I_{\text{incident}}(x,y)\right|]$ to show the displacements. From the third row of Supplementary Figure 7, the displacements of the whole beams are clearer to be seen.

Supplementary Note 10. The observed beam shifts at different wavelength

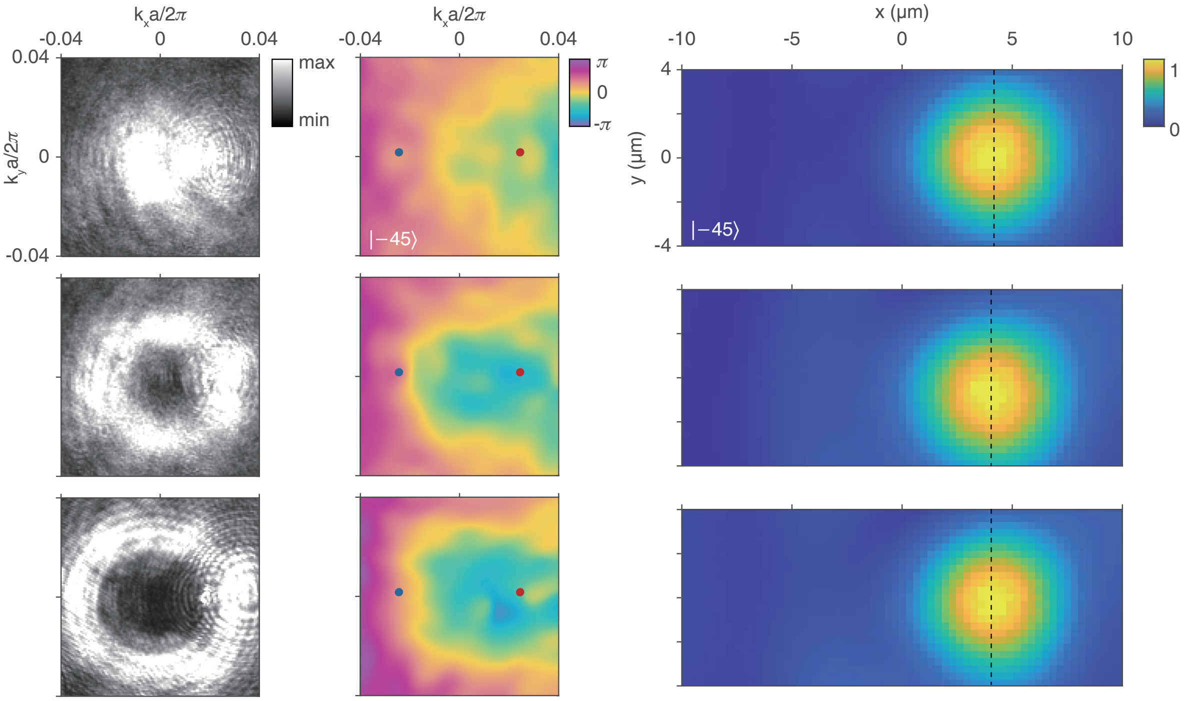

Supplementary Figure 8: **Real-space measurement of the analyzed transmitted beam at different wavelengths with a |−45⟩-polarized incidence.** Different rows correspond to different wavelengths, 817 nm, 819 nm, and 821 nm correspondingly. **The first column** shows the momentum-space intensity profiles of the cross-polarization-converted beams at different wavelengths. It turns from an elliptical spot into a ring. **The second column** shows the momentum-space phase distribution of the analyzed beams. The difference in the phase distributions only comes from the resonant phase, which will not affect the phase gradients determining the beam shifts. **The third column** shows the real-space beam profiles after passing through the analyzer. According to the slightly lower geometric phase gradients, the beam shifts are slightly smaller for larger wavelengths.

We measure the analyzed beam profiles at different incident wavelengths (817 nm, 819 nm, and 821 nm) both in momentum space and real space, plotted as Supplementary Figure 8. Since the momentum-space profile of the beam turns into a ring when the wavelength becomes larger and the average phase gradient obtained by the beam becomes slightly lower, the beam shift at a larger wavelength is slightly smaller. Even so, the difference is not obvious.

Supplementary Note 11. Modulating the polarization-dependent lateral shifts by controlling the symmetry-breaking factor: experiment

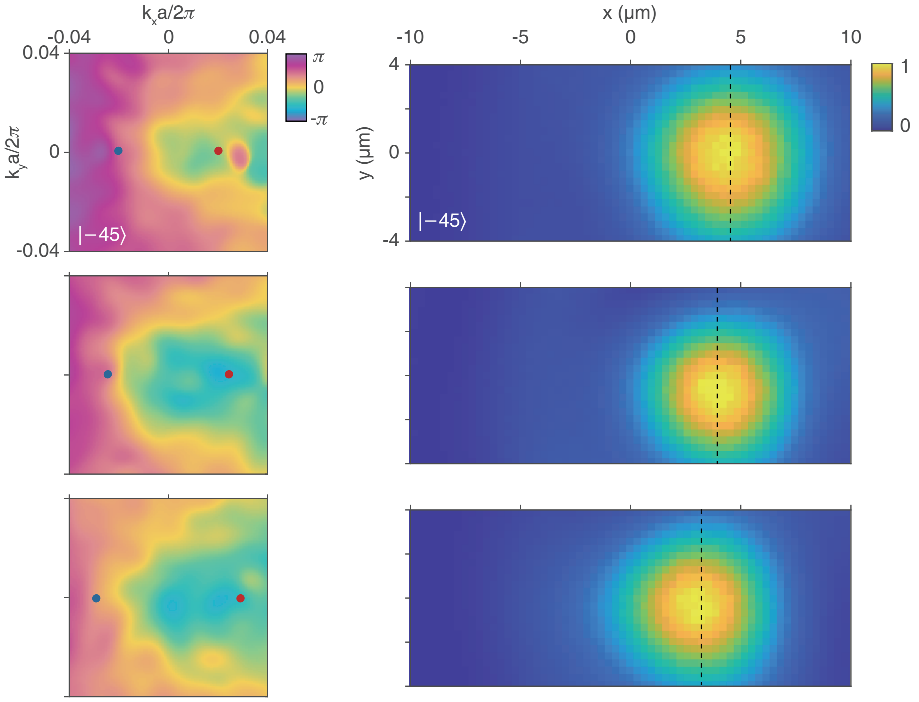

Supplementary Figure 9: **Real-space measurement of the analyzed transmitted beam with different symmetry-breaking factors with a |−45⟩-polarized incidence.** Different rows correspond to different samples. **The first column** shows the momentum-space phase distribution of the analyzed beams. We marked the positions of the C-points which are obtained from transmittance spectra under circularly-polarized incidence. The average phase gradient becomes lower from top to bottom. **The second column** shows the real-space beam profiles after passing through the analyzer. Correspondingly, the beam shift becomes smaller from top to bottom. The displacements of the beam maxima are (4.6, 3.9, 2.9) microns.

As discussed in **Supplementary Note 3**, **Note 4**, **Note 7**, the displacement of beam shift is determined by the momentum-space phase gradient, and such a gradient is related with the symmetry-breaking factor of the structure. We fabricate more samples with different symmetry breaking factors and try to experimentally observe different beam shifts by controlling the symmetry-breaking factor. In the experiments, we cannot give an explicit symmetry-breaking factor. But from the positions of the C-points, the phase maps and accordingly the real-space beam profiles, we can clearly see the correlation between the symmetry breaking and the displacements. The results are plotted in Supplementary Figure 9.

Supplementary References

[1] Wenzhe Liu, Bo Wang, Yiwen Zhang, Jiajun Wang, Maoxiong Zhao, Fang Guan, Xiaohan Liu, Lei Shi, and Jian Zi. Circularly polarized states spawning from bound states in the continuum. *Physical review letters*, 123(11):116104, 2019.

[2] Bo Wang, Wenzhe Liu, Maoxiong Zhao, Jiajun Wang, Yiwen Zhang, Ang Chen, Fang Guan, Xiaohan Liu, Lei Shi, and Jian Zi. Generating optical vortex beams by momentum-space polarization vortices centred at bound states in the continuum. *Nature Photonics*, 14(10):623–628, 2020.
